# A Phosphorylation Tag for Uranyl Mediated Protein Purification and Photo Assisted Tag Removal

**DOI:** 10.1371/journal.pone.0091138

**Published:** 2014-03-05

**Authors:** Qiang Zhang, Thomas J. D. Jørgensen, Peter E. Nielsen, Niels Erik Møllegaard

**Affiliations:** 1 Department of Cellular and Molecular Medicine, Faculty of Health Sciences, University of Copenhagen, Copenhagen N, Denmark; 2 Department of Biochemistry and Molecular Biology, University of Southern Denmark, Odense M, Denmark; Russian Academy of Sciences, Institute for Biological Instrumentation, Russian Federation

## Abstract

Most protein purification procedures include an affinity tag fused to either the N or C-terminal end of the protein of interest as well as a procedure for tag removal. Tag removal is not straightforward and especially tag removal from the C-terminal end is a challenge due to the characteristics of enzymes available for this purpose. In the present study, we demonstrate the utility of the divalent uranyl ion in a new procedure for protein purification and tag removal. By employment of a GFP (green florescence protein) recombinant protein we show that uranyl binding to a phosphorylated C-terminal tag enables target protein purification from an *E. coli* extract by immobilized uranyl affinity chromatography. Subsequently, the tag can be efficiently removed by UV-irradiation assisted uranyl photocleavage. We therefore suggest that the divalent uranyl ion (UO_2_^2+^) may provide a dual function in protein purification and subsequent C-terminal tag removal procedures.

## Introduction

Purification of proteins can be simplified by employment of affinity tags fused to either the N or C-terminal end of the proteins [1], [2]. Although some tags may have positive effects, such as increasing solubility of the protein or promoting correct folding [3]–[5] more often tags adversely effect protein activity [6]–[8]. Thus, in most cases tag removal is a crucial requirement before subsequent use of a protein [9], [10]. Tags may be removed by chemical treatment, such as cyanogen bromide cleavage [11] [12]. However, chemical cleavage requires harsh solvent conditions and there is a high risk of side effects such as protein denaturation together with cleavage and modifications of amino acids within the protein [13], [14]. More widely used processes for tag removal take advantage of enzymatic treatment using naturally occurring proteolytic endopeptidases (thrombin, enterokinase, factor Xa or TEV protease) [4], [15]–[17]. The disadvantage using endopeptidases is risk of cleavage at natural sites within the target protein as well as inefficient cleavage of some fusion proteins [9], [18], [19]. Likewise, exoproteases can be used to remove tags, as exemplified by the TAGzyme system based on dipeptide aminopeptidase I, which removes amino acids from the N-terminal end until a dipeptide stop signal is reached [20]. In general, employment of enzymatic tag removal is not straightforward, since both exo- and endopeptidases may result in non-specific as well as inefficient cleavage of the tag leaving several amino acids on the target protein.

In general, endopeptidases are useful for removal of tags at the N-terminal of the protein, because these enzymes cleave C-terminal to the recognition sequence [4]. However, C-terminal protein tags can be advantageous; for instance when tags in the N-terminal end interfere with a signal peptide and thus secretion of the protein. Use of endopeptidases is possible but as noted such enzymes will leave amino acids from the tag in the C-terminal the protein, because the scissile peptide bond is C-terminal relative to the recognition sequence. In principle, carboxypeptidases may be used for removing C-terminal tags. However, the carboxypeptidases are likewise highly dependent on the amino acid sequence context in the tag and in general it is not possible to obtain a protein with the native C-terminal end [21], [22]. Consequently, a need exists for development of new strategies for efficient and specific removal of tags especially from the C-terminal end of proteins.

We have previously shown that proteins can be selectively and efficiently photocleaved at phosphorylated serines by the uranyl (VI) ion (UO_2_^2+^) most likely mediated by a very strong uranyl interaction with phosphates and subsequent photooxidative cleavage [23]. Indeed phosphorylation of a specific amino acid within a calmodulin peptide highly increased affinity for uranyl due to specific phosphate uranyl interaction [24]. Thus, we speculated whether such strong phosphate binding and selective photocleavage at phosphoserines by uranyl could be exploited for affinity purification and tag removal in protein purification procedures. In order to evaluate this hypothesis, we fused a peptide tag, which is a substrate for casein kinase II, to the C-terminal end of green fluorescent protein (GFP) as a model protein. When phosphorylated the tag provides a very strong binding site for the uranyl ion and by employment of immobilized metal ion affinity chromatography (IMAC) and photocleavage, we show that both protein purification and phospho-tag removal is feasible employing this principle.

## Results and Discussion

**Figure 1:**
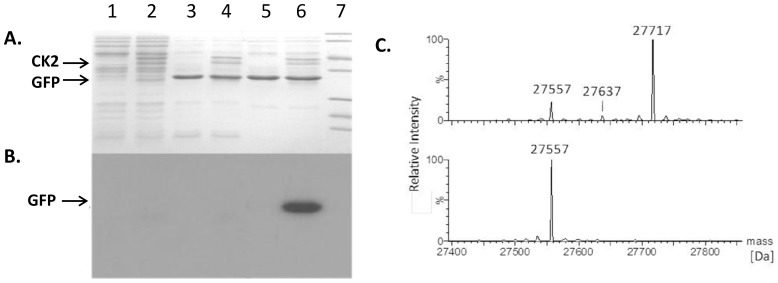
Analysis of phosphorylation of GFP28 with CK2. A and B: SDS PAGE analysis. Coomassie brilliant blue staining (A) and x-ray film (B). The lane numbers corresponds both to A and B. Lanes 1, 2: Protein extract without GFP; Lanes 3, 4: Protein extract with GFP0 (no tag); Lane 5, 6: Protein extract with GFP28. Lanes 2, 4 and 6 is treated with CK2 (in B the radioactive labeling is performed with ^32^P-ATP). Lane 7: markers (116, 66, 45, 35, 25, 18 and 14 kDa). C: ESI-MS spectrum showing GFP28 phosphorylation. Cell lysates containing GFP28 before (lower spectrum) and after treatment with CK2 (upper spectrum).

Several challenges need to be addressed in order to construct recombinant proteins with uranyl cleavable phosphorylation tags. First of all the kinase-based phosphorylation of the tag has to be efficiently and specifically taking place at the tag. Next, the subsequent proteolytic removal of the tag needs to be specific, avoiding cleavage within the protein. Finally, tag removal must also be efficient. In principle, the tag could be positioned at both the N- or the C-terminal end of the protein. However, since we have previously found that uranyl cleavage takes place N-terminal to a phosphorylated serine in bovine β-casein, the recombinant protein in the present study was constructed with the tag positioned at the C-terminal end of GFP [23]. In the search for an appropriate kinase we focused on kinases, that phosphorylate the very N-terminal amino acid in the kinase recognition sequence. This significantly limits the number of choices. However, the commercially available bovine casein kinase II (CKII) was chosen due to the consensus substrate sequence SXXE/D with an N-terminal serine as the target for phosphorylation [25]. A GFP recombinant was constructed with the C-terminal tag SSDDDGGGGGG (GFP28). This tag is designed to comprise high affinity for the uranyl ion via three aspartic acids and the two serines as potential phosphorylation sites. In order to simplify mass analysis a stretch of glycines was positioned at the C-terminal end of the tag. For comparison GFP without the tag (GFP0) was used. Protein extracts were phosphorylated by CKII without prior purification of the target protein. Protein expression of the GFP construct was found not to be affected by the tag of GFP28 (compare lanes 3–4 with lanes 5–6, Figure 1A). Phosphorylation was detected by ^32^P radioactive labeling using γ−^32^P-ATP, and from the results presented in Figure 1A (lanes 2, 4 and 6) we conclude that only GFP28 with the C-terminal tag was efficiently phosphorylated (lane 6, Figure 1B). Thus, although two potential recognition sites for CKII exist within the GFP sequence (^2^SKGE^5^, ^100^SFKD^103^), no detectable phosphorylation at these positions was observed (compare lane 4 and 6, Figure 1B). Potentially phosphorylation may likewise take place within the bacterial proteome. However, we did not observe any other phosphorylations than in the tag. In this regard, it has been shown that hydrophobic and basic residues close to the serine are strongly disfavored in known CKII substrates, in particular at the C-terminal position to the serine (e.g., there are virtually no known substrates having a basic residue in the X positions in the consensus sequence: SXXD/E) [26]. Interestingly, the two aforementioned potential recognition sites for CKII (i.e., SKGE & SFKD) have a lysine residue at the disfavored position which would explain why these sites remain non-phosphorylated [26]. However, another contributing factor to the absence of phosphorylations is steric inaccessibility that occurs when the consensus sequence is situated within a stable conformation that prevents access of the kinase. In all recombinant protein expression strategies the efficient expression of the target protein is crucial. Phosphorylation at potential CKII sites within the bacterial proteome was not detected, which could be due to the low expression of these proteins relative to the strong expression of GFP. Next, the phosphorylated protein was analyzed by electrospray ionization mass spectrometry (ESI-MS) in order to characterize phosphorylation in the tag (Figure 1C). Phosphorylation results in a protein of mass 27717 corresponding to a mass increase by 160 Da upon CKII treatment as compared to GFP28 (27577 Da). Thus, both serine residues in the tag must be phosphorylated. It is noted that a minor peak of mass 27637 is also observed corresponding to a mono-phosphorylated protein. The phosphorylation of the protein is extensive, yet not total since a peak corresponding to non-phosphorylated GFP28 is still observed after CKII treatment.

**Figure 2:**
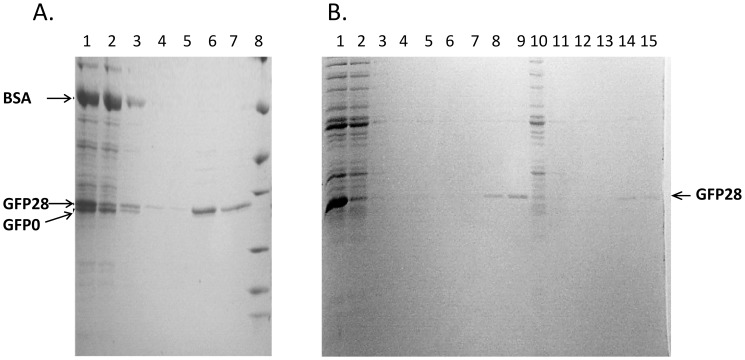
Purification of GFP28 from *E. coli* protein extracts by uranyl-NTA agarose beads. SDS PAGE analysis by Coomassie brilliant blue staining. A.1: protein mixture (cleared cell lysate with GFP28, BSA and GFP0 treated with CK2); 2: supernatant after the beads are precipitated; 3–5: supernatant from washing steps; 6–7: eluates and 8: markers (see Figure 1). **B.** protein mixture (cleared cell lysate with GFP28 treated with CK2); 2: supernatant after the beads are precipitated; 3–7: supernatant from washing steps; 8–9: eluates. Second round of purification: 10: supernatant after the beads are precipitated; 11–13: supernatant from washing steps; 14–15: eluates.

Metal ions such as Ga^3+^ or Fe^3+^ complexed to nitrilotriacetic acid (NTA) or iminodiacetic acid (IDA) sepharose have been used for enrichment of phosphopeptides in order to determine phosphorylation sites by mass spectrometry [27], [28]. However, employment of metal ions has not previously been used in protein purification procedures by the use of a phosphorylated tag. In order to examine specificity for phosphorylation tagged GFP, two protein extracts from *E. coli* expressing GFP without the tag (GFP0) and GFP with the tag (GFP28) were mixed and enzymatically phosphorylated. Despite the small difference in molecular mass (eleven amino acids), GFP0 and GFP28 show different mobility by SDS PAGE (lane 1, Figure 2A). It has previously been demonstrated that uranyl cleavage of non-modified bovine serum albumin (BSA) takes place upon irradiation [29]. Therefore, BSA was added to the *E. coli* protein extract as an internal non-phosphorylated control, and capture was done using uranyl-NTA agarose beads, which was found to be the most efficient matrix in terms of binding and elution of the phosphorylated protein (data not shown). After binding of the proteins the uranyl loaded beads with captured proteins were isolated by centrifugation. As expected, the relative amount of phosphorylated GFP28 in the supernatant was significantly lower than that of GFP0, BSA and all the other proteins in the cell lysate (lane 2, Figure 2A) (The GFP28 in the supernatant may correspond to non-phosphorylated GFP28 as complete phosphorylation was not achieved or phosphorylated GFP28 not captured by the uranyl loaded beads). It is noted that the enrichment of GFP could be detected by the green fluorescence of the protein (data not shown). From this result we infer that the phosphorylated tag of protein GFP28 has selectively bound to the uranyl loaded beads. After washing of the beads (lane 3, 4 and 5) the phospho-tagged GFP28 could be eluted with phosphate buffer (Figure 2, lane 6 and 7). The high expression of GFP compared to all other proteins in the extract may not quantitatively reflect the selectivity for the phosphorylated target protein. Thus, in order to demonstrate the selectivity for phosphorylated GFP28 the supernatant from a first round of binding (Figure 2B lane 2) was loaded on a second batch of uranyl beads (Figure 2B). This fraction contains most other bacterial proteins together with a minor fraction of the GFP recombinant protein (compare lane 1 and 2). Also under these conditions the phosphorylated GFP binds selectively to the beads (lane 14 and 15) even though other proteins from the cells are present at similar or even higher concentrations (compare lane 2 and 10). Therefore, endogenously phosphorylated proteins must be present at low concentrations since these proteins do not co-purify. This result is in accordance with the known low abundance of phosphorylated proteins in bacteria compared to eukaryotic cells [30]. Furthermore, although several potential phosphorylation sites are found in the E. coli proteome, it seems as these potential CKII phosphorylation sites are not efficiently phosphorylated. Selective purification of recombinant phospho-tagged GFP is clearly achieved, demonstrating that uranyl may be used in immobilized metal ion affinity chromatography (IMAC) for enrichment of phospho-tagged proteins. Furthermore, this finding makes uranyl, analogously to Ga^3+^ and Fe^3+^, an interesting candidate in IMAC for analysis of the phosphoproteome.

**Figure 3:**
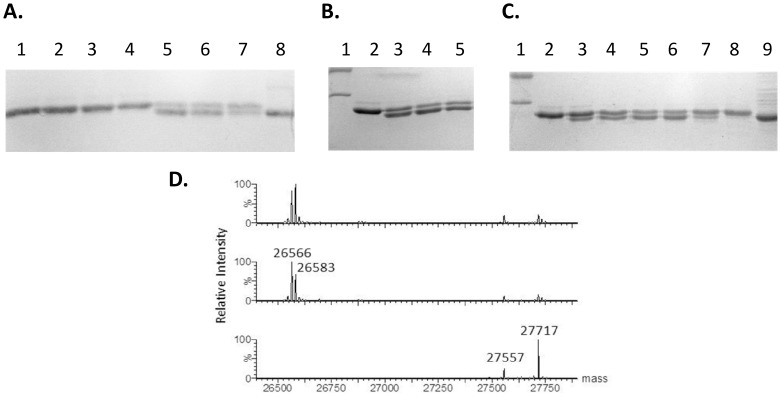
Uranyl photocleavage of phosphorylated GFP28 analyzed by SDS PAGE and ESI-MS. **A.** Lane 1–4: samples incubated for 30 minutes without irradiation in the presence of 0, 25, 50, 100 and 200 µM uranyl, respectively; Lane 5–7: samples irradiated for 30 min at 320 nm in the presence of 25, 50 and 100 µM uranyl, respectively; lane 8: cell lysate with GFP0 (no tag) **B.** Cleavage on ice. Lane 1: markers (35 and 45 kDa); lane 2: no irradiation, lane 3–5: irradiation on ice for 30 min in the presence of 25, 50 and 100 µM uranyl, respectively. **C.** Effect of pH. Lane 1: markers (35 and 45 kDa), lane 2: no irradiation; lane 3–8: cleavage in the presence of 25 µM uranyl at pH 9, 8.5, 8, 7.2, 6 and 4.5, respectively; lane 9: cell lysate with GFP0 (no tag) **D.** ESI-MS analysis of phosphorylated GFP28 cleavage. I: GFP treated with CK2; II and III: phosphorylated GFP28 irradiated (320 nm) at 50 and 100 µM uranyl, respectively.

GFP28 was used for analyzing uranyl cleavage specificity and efficiency. Both phosphorylation and cleavage were completed in a protein extract from a bacteria clone overexpressing the GFP recombinant. A uranyl dose response experiment (25, 50 and100 µM) showed that cleavage is light dependent and results in a single new band migrating below the full-length GFP28 in SDS-PAGE (Figure 3A). Furthermore, cleavage can take place on ice (Figure 3B), which may be a significant advantage to assure stability of the protein during purification. Finally, cleavage is highly dependent on pH (Figure 3C) being optimal at pH 7.2 and weak at higher and lower pH (weak cleavage at pH 6.0, 8.5, 9 and absent cleavage at pH 4.5). This probably reflects the affinity of uranyl for the phosphorylated amino acids as it was previously shown that uranyl binding to a phosphorylated peptide is dependent on pH with strongest binding observed at pH 7 [24]. The appearance of a cleavage product from GFP28 migrating exactly at the position of the native GFP (Figure 3) suggests that the tag is efficiently cleaved off. In order to identify the cleavage site, the uranyl-UV treated sample was analyzed by ESI-MS (Figure 3D). The MS results revealed two cleavage products of 26566 Da (major) and 26583 Da (minor)), respectively. The mass difference (1151±1 Da) between the double phosphorylated GFP28 (27717 Da) and the major product (26566 Da) corresponds to the loss of the C-terminal sequence KS^P^S^P^DDDGGGGGG (theoretical mass difference 1167.9–18 = 1149.9 Da), where K originates from the C-terminal end of GFP. This tag cleavage is therefore removing the last lysine from GFP. A small series of different tags were tested in order to shift the cleavage sites leaving the lysine on GFP but so far without success. It was not possible to detect the C-terminal tag product by mass spectrometry. Another minor cleavage product, which has 17±1 Da higher mass has been demonstrated by MS/MS (*data not shown*) to result from oxidation of a methionine six amino acids from the C-terminal end, which indicates that side reactions may appear if methionine is present close to the tag. We have also analyzed a peptide where the methionine was changed to alanine and in that case we did not observe any oxidation (*data not shown*). Alternatively, methionine oxidation may occur during electrospray ionization [31].

We have demonstrated that the phosphate binding ability of the uranyl ion and the subsequent light assisted specific cleavage in the tag may provide a new strategy for protein purification and tag removal from the C-terminal end of recombinant proteins. Immobilized uranyl ions on NTA agarose shows a very selective and efficient purification of the GFP recombinant only if the tag is phosphorylated. The light dependent uranyl cleavage at phosphorylation sites is very selective in naturally occurring proteins, like α-casein, β-casein and ovalbumin [23]. This is corroborated by the analysis of the recombinant GFP model protein in this study. We are currently studying whether it is possible to remove the tag by irradiation while the protein is bound to the uranyl beads in order to develop a single step purification and tag removal procedure. Although the exact mechanism of cleavage has not been elucidated, the ESI-MS analysis clearly indicates that the uranyl cleavage of the peptide bond leaves an intact C-terminal amino acid. However, in order to identify the exact mechanism the C-terminal fragment needs to be identified. Although cleavage within the phospho-tag is very efficient, analysis of a series of other tags containing different amino acid combinations revealed that it is not straightforward to direct cleavage exactly to a desired amino acid position, for instance at the very C-terminal end of the protein and not at the second amino acid before the C-terminal as in this study using GFP. Thus, further studies are needed to elucidate and understand the sequence context dependence of the cleavage.

## Materials and Methods

### Construction of Tag Expressed C-terminal of GFP

Plasmid p369 expressing GFP [32] was used as template for PCR. The designed oligonucleotides including the tags were used as primers for PCR. As a result the tag is expressed C-terminal to GFP. T4 Polynucleotide Kinase (Fermentas) was used for phosphorylating the primers and vector DNA prior to ligation. The new plasmids were constructed by PCR using AccuPOL™ DNA Polymerase (Ampliqon). In order to get rid of the template we used Dpn1 (Fermentas), which cleaves the DNA by recognizing the sequence GA↓TC containing N^6^-methyladenine [33]. 1 µl Dpn1 was added directly to the PCR product, and incubated for at least 1 hour. T4 DNA Ligase (Fermentas) was used for circularization of the PCR products. 50 µl competent cells were transformed by adding 2 µl of 10 ng/µl plasmid DNA. Preparation of selected plasmids was done by using GeneJET™ Plasmid Miniprep Kit (Fermentas).

### GFP Induction and Extraction from *E. coli*

20 ml LB medium was inoculated with *E. coli* with the plasmid and supplied with 0.1% arabinose and 100 µg/ml ampicillin and incubated overnight. The cell culture was centrifuged at 8000×g for 5 min. Cells were washed by adding 20 ml water followed by centrifugation at 12000 g for 10 min. 300 µL B-PER was added and supplemented with 1 µL DNase I and 2 µL 1 mg/ml RNase A and incubated for 60 min. Finally, the cell lysate was purified by centrifugation for 30 min at 20000×g.

### Phospho-tagged GFP Purification by uranyl-NTA Beads

One volume NTA agarose was washed with 2 volume water. Subsequently, the beads were loaded with 1 volume 100 mM uranyl. The beads were washed twice with two volume water and twice with two volume of equilibration buffer. Finely, the beads were supplied with one volume 20% ethanol and stored at 4°C. For protein purification two volumes of uranyl-NTA agarose beads were added two volumes of binding buffer. The samples were added and binding were taking place with gentle agitation at 4°C for 60 min. The beads were washed with six volumes washing buffer followed by elution with one volume elution buffer.

### Phosphorylation and Uranyl Photo Cleavage of GFP28

Cell lysate is mixed with 1×casein kinase reaction buffer, 200 µM ATP and casein kinase II. In the experiment of Figure 1B demonstrating the specific phosphorylation of the tag ^32^P-ATP was used. The samples were incubated at 30°C. The reaction mixture was purified by running the samples through PD SpinTrap G-25. Uranyl solution was prepared by dissolving UO_2_(NO_3_)^2^ in water to a final concentration of 100 mM. Reaction condition was: 20 mM Tris-HCl, pH 7.2 and 0.05% NP40 if nothing else is indicated. Uranyl solution of a series of concentrations was added just before UV irradiation in different containers on ice or at RT for 5 to 60 min.

### SDS PAGE and Coomassie Blue Staining

SDS-Polyacrylamid gels were prepared by standard procedure. The samples were mixed with 6×SDS loading buffer (4 ml 1 M pH 6.8 Tris-HCl mixed with 1.2 g SDS until clear, 0.93 g DTT, 250 µl Bromophenolblue in glycerol and 4 ml glycerol.), heated at 75°C for 5 min and 4°C centrifuged for 1 min at max speed. The electrophoresis was performed in Mini Protean 3 Cell (BioRad) apparatus with running buffer (25 mM Tris-base, 0.192 M glycin, 0.05% SDS) in the inner and outer chambers. The gels were run for 1.7 hr at 200 V. The gels were stained overnight in Coomassie Blue Stain (0.1% w/v Coomassie Brilliant Blue R250, 40% ethanol, 10% acetic acid) and destained in destain solution (25% ethanol, 8% acetic acid).

### Mass Spectrometry and Data Analysis

Positive ion electrospray ionization (ESI) mass spectra were acquired on a Waters Synapt G1 quadrupole time-of-flight mass spectrometer equipped with a megaflow ion source. The ion source settings were: capillary voltage 3.5 kV, sampling cone voltage 26 V, extraction cone 2.6 V, ion source block temperature 70°C, desolvation gas (N_2_) flow 500 L/h (150°C). The instrument was calibrated using apomyoglobin. Mass spectra were acquired for the mass range m/z 300–2000 with a detector (MCP) voltage of 1700 V to increase the signal-to-noise ratio for multiply charged ions. MS spectra were processed using Masslynx software (v. 4.1) and spectrum deconvolution was carried out with the maximum entropy algorithm (MaxEnt 1) included in this software. The protein samples were desalted by reversed-phase chromatography using a Waters MassPREP Micro Desalting Column. Gradient and desalting flows were provided by a Waters nanoACQUITY UPLC pump and a Dionex Ultimate 3000 HPLC pump. Desalting was carried out with 0.23% formic acid (v/v) (Solvent A). Elution from the colum was carried out with a short acetonitrile gradient.
